# A Novel Surgical Reconstruction Technique in the Management of Chronic Ulnar Collateral Ligament Tears with Volar Subluxation

**DOI:** 10.5152/eurasianjmed.2022.22024

**Published:** 2022-06-01

**Authors:** Ömer Ayık, Mehmet Demirel

**Affiliations:** 1 Department of Orthopedics and Traumatology, Atatürk University Faculty of Medicine, Erzurum, Turkey; 2 Department of Orthopedics and Traumatology, İstanbul University İstanbul School of Medicine, İstanbul, Turkey

**Keywords:** Ulnar collateral ligament injury, thumb dorsal joint capsule, volar subluxation, first metacarpophalangeal joint, UCL reconstruction, reconstruction of the dorsal joint capsule

## Abstract

**Background:** We hypothesized that ulnar collateral ligament reconstruction is inadequate for metacarpophalangeal joint stabilization in chronic ulnar collateral ligament injuries with volar subluxation due to dorsal joint capsule injury. We consecutively performed both ulnar collateral ligament and dorsal joint capsule reconstruction to treat patients with a chronic ulnar collateral ligament tear with volar subluxation. This study aimed to present our preliminary results and experience with this technique in managing such cases.

**Materials and Methods:** In this retrospective study, 7 patients (6 males, 1 female) who underwent surgical reconstruction of both ulnar collateral ligament and dorsal joint capsule reconstruction for the treatment of chronic ulnar collateral ligament injuries with volar subluxation were included. The mean age was 31 (range = 20-39) years, and the mean follow-up was 15.5 (range = 12-20) months. Several clinical and radiological data were recorded.

**Results:** The mean Visual Analogue Scale score significantly improved from 5.7 (range = 5-8) to 0.57 (range = 0-1) (*P* < .001). The mean quick- Disabilities of the Arm**,** Shoulder and Hand was significantly improved from 31.8 (range = 27.3-38.6) preoperatively to 3.2 (range = 0-6.8) at the final follow-up (*P* < .001). The mean preoperative extension deficit decreased from 18.5° (range = 10°-25°) to 0° (range= 0°-0°) at the final follow-up (*P* = .022). The mean preoperative flexion deficit increased from 10.7° (range = 0°-20°) to 31.4° (range = 25°-35°) postoperatively (*P* = 0.034). The mean key-pinch strength significantly increased from 33.2% (range = 27-37) preoperatively to 10.2% (range = 6-14) at the final follow-up assessment (*P* < .001). The mean hand grip strength significantly increased from 18.8% (range = 15-23) preoperatively to 6.4% (range = 6-14) at the final follow-up assessment (*P* < .001).

**Conclusion:** With encouraging short-term clinical outcomes and a lower complication rate, surgical reconstruction of both ulnar collateral ligament and dorsal joint capsule seems to be a safe and effective surgical technique in the management of chronic ulnar collateral ligament tears with volar subluxation.

**Main Points**

- The ulnar collateral ligament (UCL) constitutes the primary stabilizer of the thumb against valgus deformity forces.
- The UCL tears concur dorsal joint capsule (DJC) and result in volar–dorsal instability.
- To date, no surgical technique has been described to reconstruct DJC in addition to UCL.
- Reconstruction of UCL and DJC tears can be an effective surgical technique in managing chronic UCL tears with volar subluxation.

## Introduction

The main component of the first metacarpophalangeal joint (MCPJ) stabilization is supportive soft tissue along with the bone structure, which has a minor role. These supportive structures consist of both static stabilizers such as ligamentocapsular structures and dynamic stabilizers such as intrinsic and extrinsic muscles. The ulnar collateral ligament (UCL) constitutes the primary stabilizer of the thumb against valgus deformity forces such as pinch and grip forces.^1^

**Figure 1.**
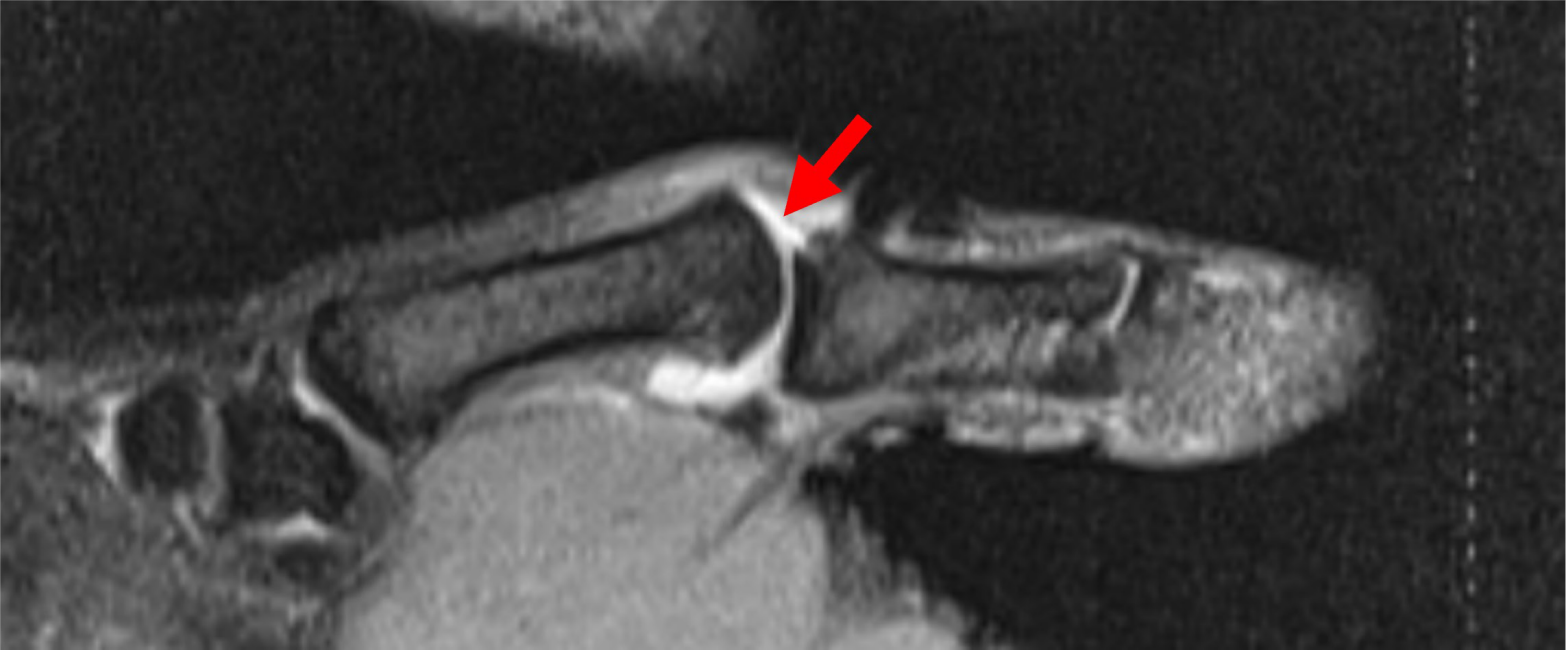
The sagittal magnetic resonance imaging scan shows chronic ulnar collateral ligament tear of the first metacarpophalangeal joint. Note that volar subluxation of the joint secondary to tear of the dorsal joint capsule (red arrow).

Fall on an outstretched hand while the thumb is abducted or sports injuries, especially during skiing, may cause UCL tears. These tears will result in valgus instability in the first MCPJ. Occasionally, these traumas concur with dorsal joint capsule (DJC), radial collateral ligament (RCL), or volar plate (VP) injuries. Mainly, DJC tears result in volar–dorsal instability. In concomitant UCL and DJC injuries, RCL acts as a pivot due to strong contraction of flexor and thenar muscles, and thus, supination and volar subluxation deformity at the first MCPJ will occur (2) (Figure 1). If the joint luxation and instability are left untreated, osteoarthritis occurs in the long term.^2,3^

To date, some surgical approaches for UCL tears with volar subluxation have been defined in the literature. Mostly, satisfactory results of primary repair for acute trauma of UCL and DJC have been noted. Although there is no consensus on chronic injuries, the widespread opinion is that UCL reconstruction is sufficient. Nevertheless, those studies have low evidence levels, a small sample size, and inadequate detection power.^3,4^

Based on our clinical experience, we hypothesized that UCL reconstruction is inadequate for MCPJ stabilization in chronic UCL injuries with volar subluxation since the MCPJ is grossly unstable in more than one direction due to DJC injury. Therefore, in our opinion, the treatment should include DJC reconstruction in addition to the UCL reconstruction. We consecutively performed both UCL and DJC reconstruction simultaneously to treat patients with a chronic UCL tear with volar subluxation. This study aimed to present our preliminary results and experience with this technique in managing such cases.

## Patients and Methods

Eight patients with chronic UCL injuries and volar subluxation who had undergone surgical reconstruction of both UCL and DJC reconstruction between 2018 and 2020 in a single tertiary institution were retrospectively reviewed. Inclusion criteria for the study were

1. verification of volar subluxation with lateral x-ray or sagittal magnetic resonance imaging (MRI);
2. a minimum follow-up period of 12 months;
3. complete medical and radiological data; and
4. patients with a mental status that would comply with the treatment.

### Exclusion criteria included

1. head and capsule structure suitable for primary repair (especially cases earlier than 4-6 weeks);
2. additional RCL or volar plate injury;
3. additional thumb phalanx (including bone avulsion) or metacarpal fractures;
4. MCPJ arthrosis confirmed by x-ray; and
5. MCPJ contracture in physical examination.

All the patients were evaluated based on the above eligibility criteria. After excluding 1 patient who was lost to follow-up, the remaining 7 patients (6 males, 1 female) who met the inclusion criteria were invited to a final follow-up examination. Ethics Committee of Erzurum RERH approved this study before data collection (2022/02-06), and all patients gave informed consent.

### Preoperative Data

The mean age was 31 (range = 20-39) years, and the mean follow-up was 15.5 (range = 12-20) months. There were 5 right and 2 left thumbs, with the dominant hand involved in 5 cases. In terms of profession, 2 patients were manual workers, 2 were white-collar workers, 1 was a farmer, 1 was an athlete, and 1 was a student (Table 1). Every patient had a history of irregular use of nonsteroid antiinflammatory drugs (NSAIDs) and thumb splints.

All the patients had painful thumb movement during daily life and weakness in activities requiring strength such as pinch and grip. A prominent extension deficit compared to the other side was noted. In inspection, MCPJ were more swollen, and metacarpal heads were more prominent than on the other side. In palpation, MCPJ were painful, and protuberances at the ulnar side were palpated. Valgus stress test, anterior, and posterior drawer tests were found positive compared to the other side in each patient. The mean preoperative duration of chronic wrist pain was 5.7 (range = 3-9) months. All the patients had a trauma history (5 patients had a fall history and 2 had sports injuries). None of the patients was treated with conservative treatment. Because the joints were not concentrically reduced and instability is a predisposing factor for arthrosis, all patients were treated surgically.

### Diagnosis and Radiological Assessment

Comparative bilateral standard posteroanterior and lateral x-rays in addition to the stress views were performed for all patients. All patients received MRI to verify the diagnosis, and all MRI scans were examined by a radiologist who was specialized in musculoskeletal radiology.

### Clinical and Functional Outcome Measures

Pre- and post-operative comparative first MCPJ joint stability tests, flexion–extension passive range of motion deficits, key-pinch and grip force deficits were investigated, and all the patients were graded according to the Glickel grading system.^5^ Change in pain intensity was assessed with the Visual Analogue Scale (VAS).^6^ Pre-and post-operative functional status was assessed with the Quick Disabilities of the Arm, Shoulder, and Hand (Quick-DASH).^7^

After applying adequate local anesthetics to MCPJ to achieve a painless optimal physical examination, metacarpal bone was stabilized, and a proximal phalanx valgus stress test was performed with MCPJ in 30˚ flexion and full extension. Afterward, metacarpal bone was stabilized and then the proximal phalanx anterior and posterior drawer tests were performed with MCPJ in full extension. In the valgus test, a deviation of 35° on the affected side or a difference of 15° between the affected and contralateral sides in flexion denotes a primary UCL tear. In extension, it points out a tear of the accessory part of the UCL. Volar subluxation greater than 3 mm compared to the contralateral side is usually a sign of a complete tear confirmed as positive for the anterior–posterior drawer test.^8,9^

In the assessment of range of motion (ROM), passive extension and flexion of both thumbs were first estimated by a universal standard goniometer. Subsequently, extension and flexion deficits were calculated as the difference between both sides.

The key-pinch and grip strength were measured from both affected and contralateral, healthy sides by a Jamar pinch meter and dynamometer (Jamar Hydraulic Hand Dynamometer™ and Pinch Meter, Sammons Preston, Bolingbrook, Ill, USA) preoperatively and at the final follow-up; the average value of 3 successive measurements was calculated as a percentage of the contralateral, healthy side.

Functional satisfaction was graded as very disappointed, disappointed, somewhat satisfied, satisfied, or very satisfied. Return to work was noted as a post-surgery week.

An independent hand surgeon conducted the final follow-up examination and did not attend to any of the operations. Intra-or post-operative complications such as neuritis, tendinopathy, pin-site, or wound infection were noted. Valgus instability at MCPJ and volar subluxation were defined as failure.

### Surgical Technique

**Figure 2.**
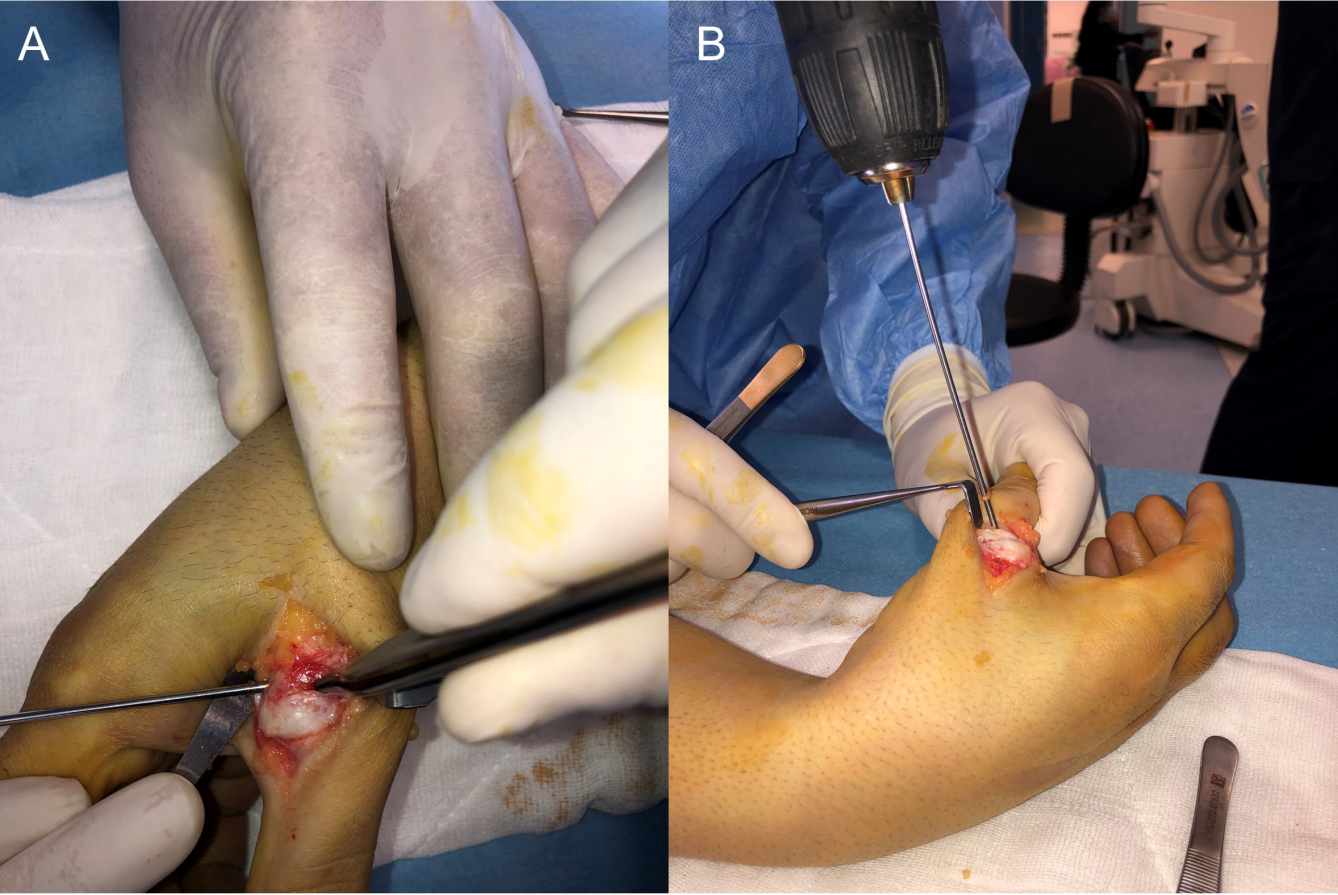
Illustration of drilling the hole on the ulnar (A) and radial (B) sides of the first metacarpal.

Under regional anesthesia, the patient was placed in the supine position on a hand table. Skin flaps were elevated with a straight midline incision after subcutaneous tissues were passed with dissecting scissors. Reconstruction was planned for the ligaments that were not suitable for primary repair. Ipsilateral-free palmaris longus graft was preferred. A 4- to 6-mm hole was drilled at the ulnar side of the metacarpal bone, located slightly dorsal to the joint center of rotation, 6-7 mm distal to the joint (Figure 2A). Another hole was drilled perpendicular to the previous one, at the center of metacarpal bone dorsally, and 2 holes were connected at the intramedullary region (Figure 2B).

**Figure 3.**
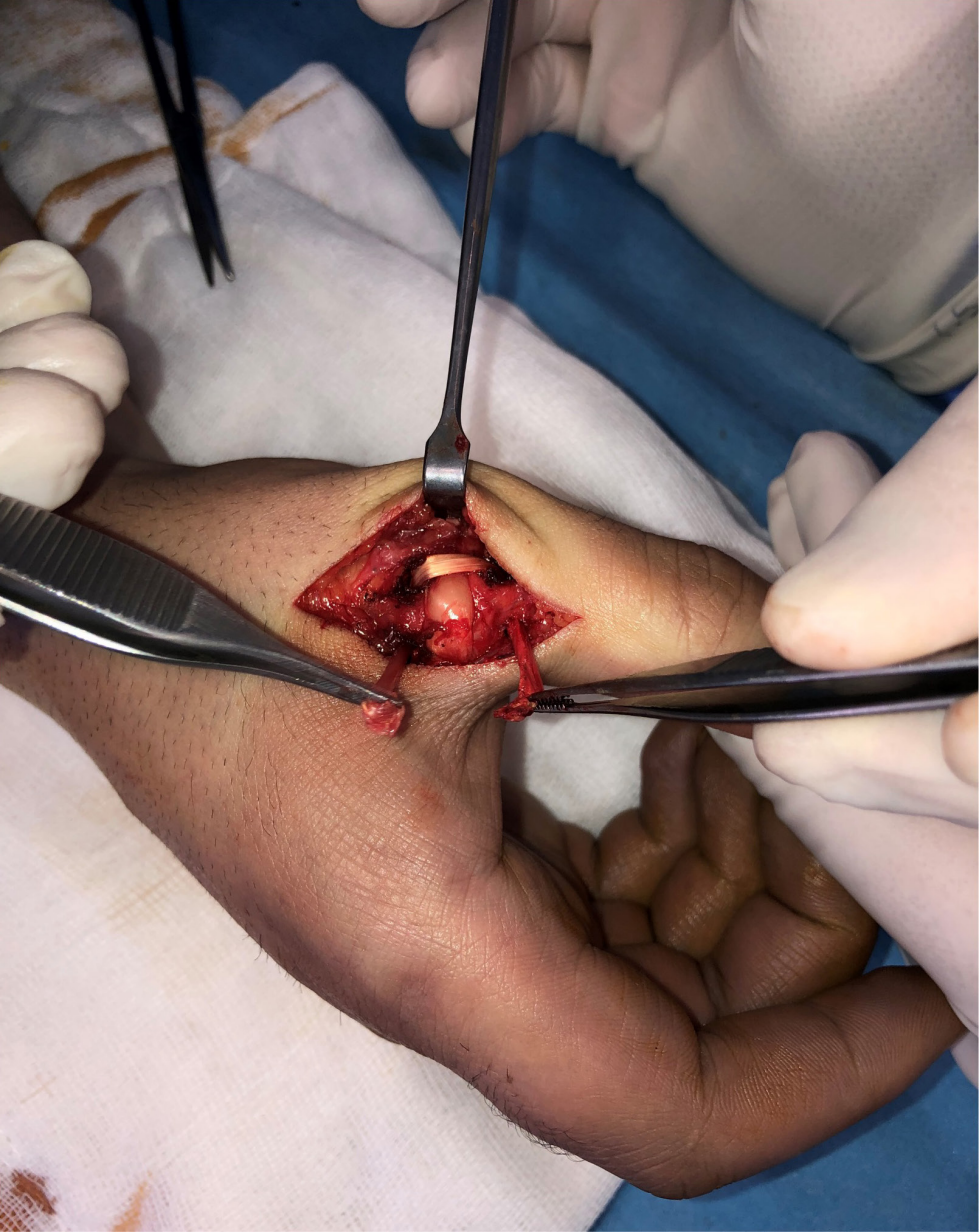
Illustration of passing the palmaris longus graft through the metacarpal and proximal phalanx holes.

To imitate the UCL orientation, another hole that is more volar to the first hole at the ulnar side of the metacarpal bone, 5-6 mm distal to the joint line, was drilled on the proximal phalanx. Lastly, another symmetric hole to the dorsal metacarpal hole was drilled on the proximal phalanx and connected in the intramedullary area similar to the one in the metacarpal bone. Free tendon graft harvested from the patient was passed through the hole on the ulnar side of the metacarpal bone and then the dorsal hole using stainless-steel wire. Afterward, the graft was passed through the hole on the dorsal side of the phalanx and then exited through the hole on the ulnar side of the phalanx (Figure 3).

**Figure 4.**
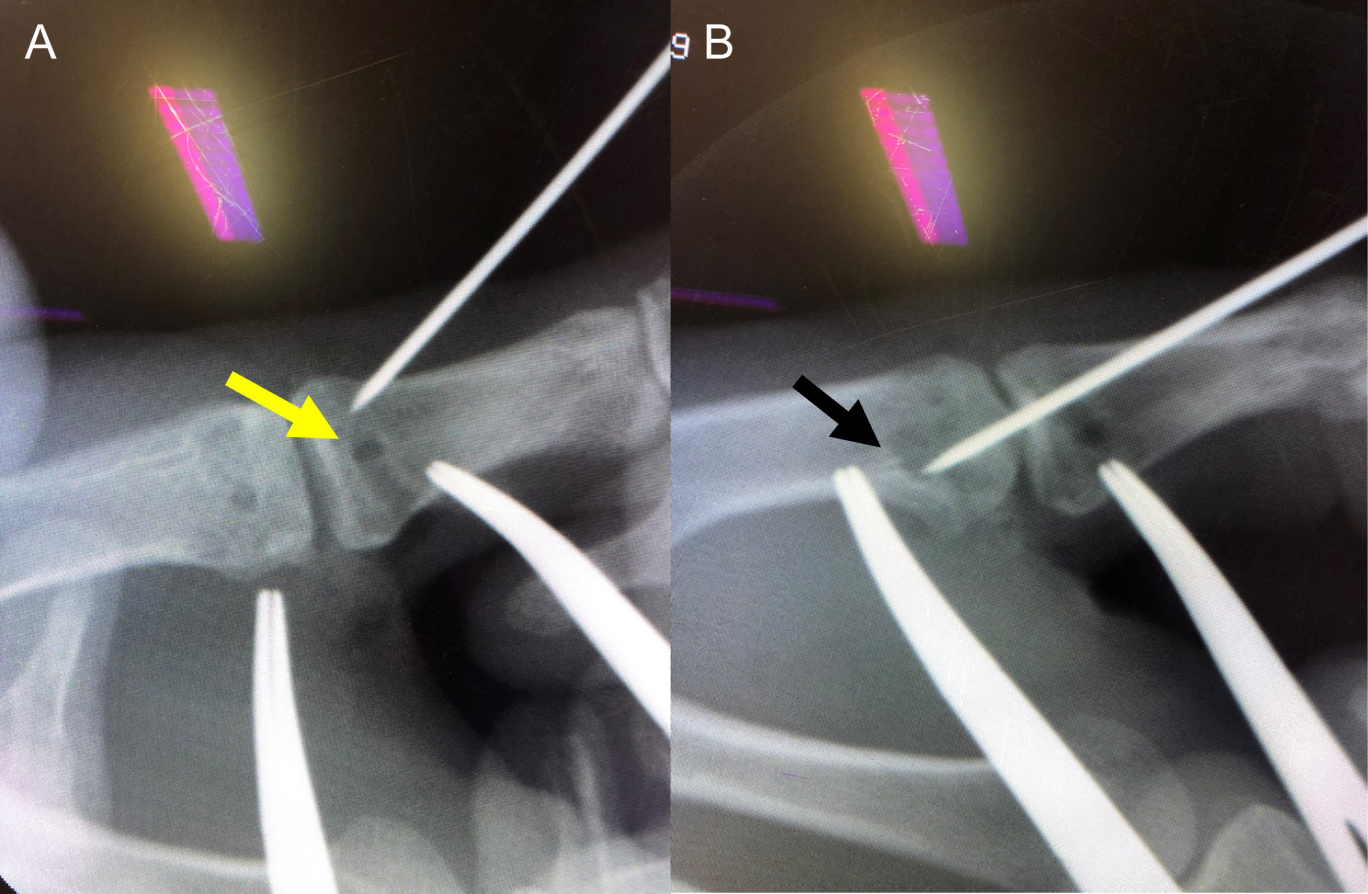
Illustration of retrograde wire fixation after reduction of the metacarpophalangeal joint in anteroposterior (A) and lateral (B) planes (yellow arrow: fluoroscopy image of dorsal holes, black arrow: the fluoroscopic image of ulnar holes).

**Figure 5.**
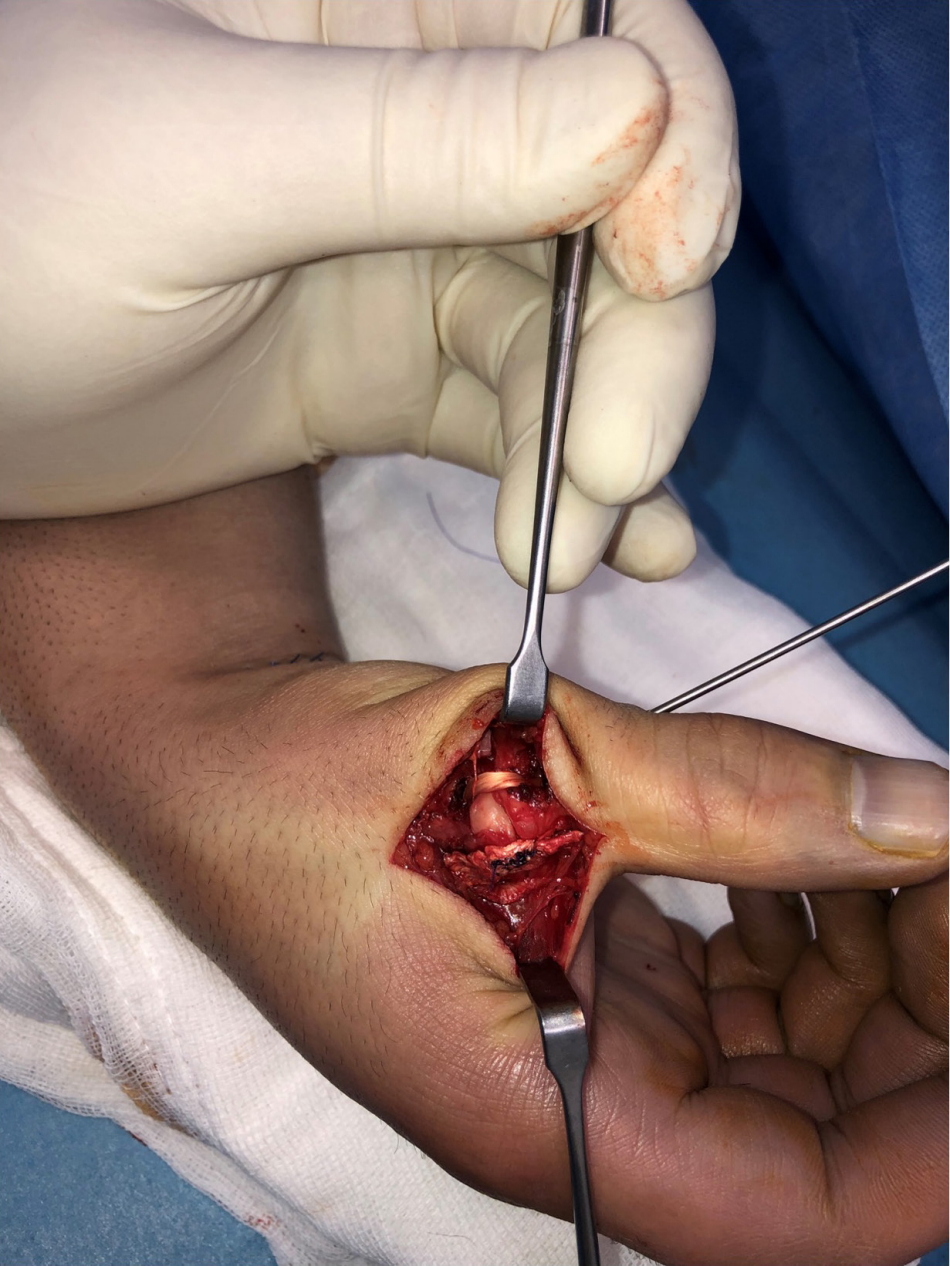
Suturing 2 sides of the graft in metacarpal and phalanx ulnar holes after pin fixation is shown.

**Figure 6.**
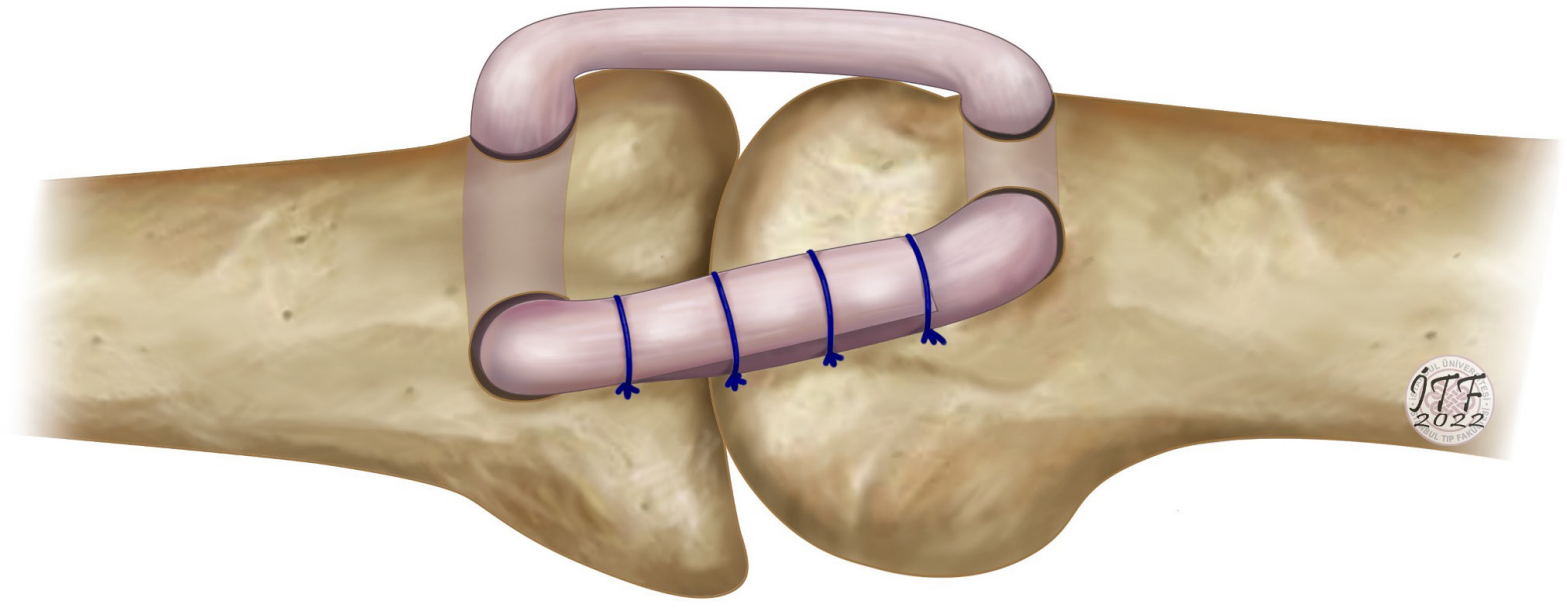
Representative illustration of the ulnar collateral ligament and dorsal capsular reconstruction.

Before the fixation of the graft, MCPJ was reduced in extension on the coronal and sagittal planes under fluoroscopy imaging. In a reduced position, MCPJ was stabilized using a 1.4-mm pin retrograde from the radial side of the proximal phalanx to the metacarpal bone (Figure 4). The tip of the pin is left outside for easier removal. Two sides of the graft in metacarpal and phalanx ulnar holes were sutured with the 4-0 slow-absorbable monofilament polydioxanone suture (PDS II 4-0, Ethicon Inc; Johnson & Johnson, Somerville, NJ, USA) (Figure 5). Figure 6 shows the representative illustration of the reconstruction. A plaster containing the wrist and allowing thumb motion was made, and the procedure was completed.

### Postoperative Management

At postoperative 1-2 weeks, the plaster was taken out, and a splint allowing thumb IP joint and wrist joint motion was rendered. After 4-5 weeks, the wire was removed at the outpatient clinic, and the splint was discarded. After the wire was removed, a control x-ray was taken to assess joint congruence. Home exercises under the supervision of hand therapists were started. First, thumb passive–active ROM exercises were allowed, and stressing was avoided.

Strengthening of the thumb was avoided until 3 months after surgery and then allowed gradually with light pinch and grip strengthening. After 3 months, a control x-ray was performed, and the patient was allowed to use the reconstructed thumb without restriction. Clinical and radiological follow-up was performed in the sixth month and the first year.

### Statistical Analysis

All statistical analyses were done using Statistical Package for the Social Sciences software v.25 (IBM SPSS Corp.; Armonk, NY, USA). A *P* value < .05 was considered significant. Test for normality of the variables was done by Shapiro-Wilk test and histogram graphics. Data are presented as “means” and “ranges (minimum to maximum).” Nonparametric paired comparisons were performed using the Wilcoxon signed-rank test.

## Results

### Radiological Findings

In radiographic assessment, all patients had >2 mm radial translation and >3 mm volar subluxation in their preoperative comparative thumb x-rays. “Sag sign” illustrates volar subluxation and rotation was positive for all patients (11). In the postoperative period, radial translation and volar subluxation were found as corrected. None of the patients had arthrosis before the surgery, and no arthrosis was observed during the postoperative period.

In MRI evaluation, for all the patients, UCL rupture signs, including the absence of normal UCL fibers spanning the MCP joint and a well-defined heterogeneous mass proximal to the metacarpal tubercle, were observed. Additionally, dorsal capsule injury and MCPJ synovitis were seen.

### Intraoperative Findings

All patients had grade 3 complete UCL tears located at distal insertion or in the distal aspect of the ligament, leaving the proximal stump intact. The dorsal capsule was injured transversely for all patients. The diagnosis of Steiner lesion was made for 3 patients for whom UCL was located at the superior aspect of adductor aponeurosis. After aponeurosis and capsule were dissected, arthritis and synovitis were observed. Joint surfaces were evaluated for chondrolysis, and none of the patients had a lesion. There was no volar plate contracture that can cause chronic volar subluxation. Two patients did not have palmaris longus (PL), a partial slip was harvested from flexor carpi radialis (FCR) for these patients.

### Clinical and Functional Outcomes

The thumb valgus stress test and anterior–posterior drawer test were positive during the preoperative period, whereas these tests were negative after the surgery.

The mean VAS score significantly improved from 5.7 (range = 5-8) to 0.57 (range = 0-1) (*P* < .001). The mean quick-DASH was significantly improved from 31.8 (range = 27.3-38.6) preoperatively to 3.2 (range = 0-6.8) at the final follow-up (*P* < .001) (Table 2).

According to the Glickel grading system, all patients were under the poor category before surgery with a mean score of 6.1 (range = 5-7). After the surgery, 4 patients were excellent, and 3 patients were good, with a mean score of 16.8 (range = 14-19) (*P* < .001) (Table 2).

In comparison with the other thumb MCPJ, the mean preoperative extension deficit decreased from 18.5° (range = 10°-25°) to 0° (range= 0°-0°) at the final follow-up (*P* = .022). The mean preoperative flexion deficit increased from 10.7° (range = 0°-20°) to 31.4° (range = 25°-35°) postoperatively (*P* = .034) (Table 2).

The mean key-pinch strength significantly increased from 33.2% (range = 27-37) preoperatively to 10.2% (range = 6-14) at the final follow-up assessment (*P* < .001). The mean hand grip strength significantly increased from 18.8% (range = 15-23) preoperatively to 6.4% (range = 6-14) at the final follow-up assessment (*P* < .001) (Table 2).

### Complications

There was no major intra- or postoperative complication. In the second week, 1 patient had a grade 1 pin-site infection and was treated with local wound treatment and antibiotics. There was no need to remove the pin early. None of the patients had stress x-ray positivity which illustrates reconstruction failure.

## Discussion

Ulnar collateral ligament injuries related to falls or sports traumas are among the most common types of injury. In the early stages of the injury, the injury can be neglected due to suboptimal physical examination in the emergency department. Because of this, patients are generally admitted to the hand surgery clinics a long time after the first incident.^10^ Shortening and scarring of ligament and capsule occur after complete UCL injury thus making primary repair unsuitable. Reconstruction is the modality of treatment for these injuries.

There is no definite time for chronicity in literature.^11^ In studies, generally, between 3 and 8 weeks, if the injured structures are suitable, the aim should be primary repair.^11^ None of the patients was suitable for primary repair in our study, and the earliest admission time was 3 months. For thumb ligament injuries that are not suitable for primary repair, certain static and dynamic surgical procedures were defined.^5,10,11^ No superiority among these procedures was recorded; nevertheless, these procedures play a crucial role in MCPJ stabilization, instability treatment, and possible arthrosis prevention.^10^

Biomechanical studies have proven that in addition to the support effect of UCL and RCL, dorsal capsule integrity is of paramount importance in preventing volar subluxation of MCPJ since flexor and thump intrinsic muscles have a more substantial pulling effect than extensors.^12,13^ Dorsal joint capsule tears that accompany some UCL tears cause dynamic valgus instability, create an additional dorsal–volar instability, and result in volar subluxation, which is a static MCPJ injury. Studies support that UCL reconstruction is sufficient for this multi-axial instability.^5,14^ However, these studies are few, have low evidence levels, have a small sample size, and do not have the adequate power to detect differences. Although Glickel reported that volar subluxation could be prevented by balancing the rotational forces in the MCPJ with the UCL reconstruction technique he defined, he also reported that the technique might sometimes be insufficient for dorsovolar instability.^11^ Pai et al^14^ illustrated that volar subluxation could be treated with primary repair even for chronic cases by advancing the scarred UCL. However, their study was conducted on only 2 cases, and larger case series are needed.^14^

For thumb function, MCPJ stability is more important than motion. Villén et al^15^ reported that in their case series of 10 patients, MCPJ valgus stress regressed from 54° preoperatively to 11° postoperatively, applying tendon graft in a triangular configuration.^15^ Our study divided these tests into 2 groups, positive and negative, and observed that preoperative positive stability tests were negative after surgery. To investigate the efficacy of the proposed technique, further studies are needed involving angular measurements.

Large reviews found that independent of the surgical technique, MCPJ reconstruction itself might cause 75%-100% motion loss.^4^ Proximal apex triangular configuration, the most similar reconstruction method to UCL anatomy, had the best results.^16^ We observed that preoperative extension loss was improved in the postoperative period (18.5% to 0% deficit compared to the contralateral side). However, preoperative flexion decreased significantly compared to the other studies (10.7% to 31.4% compared to the contralateral side). The reason for this decrease is thought to be excessively tight dorsal capsule reconstruction. In our study, we mention 1-year follow-up result. However, in different studies, it was illustrated that graft stretches slightly in the process of healing and rehabilitation after more than 2 years.^11^ We may observe a decrease in flexion deficit with a longer follow-up period.

Long-term subluxation may cause contractures for the structures that the joint is subluxated toward, such as RCL contracture secondary to radial translation or volar plate contracture secondary to volar subluxation. Some studies suggested that these contractures may inhibit MCPJ reduction, and they need to be relaxed if necessary.^14^ None of the patients had a contracture in these structures, and reduction was achieved in our study. The duration between the injury and surgery is relatively short (maximum 9 months) in our study; this may be why none of the patients had contractions. In other studies, it is common to encounter a longer injury period before admission.^14,15^ However, these studies contain patients with dynamic instability secondary to isolated UCL rupture. Volar subluxation is defined as static instability, which is more painful compared to dynamic instabilities thus leading patients to an earlier admission time.

In our study, for UCL injuries with volar subluxation, we hypothesized that UCL reconstruction would not be sufficient, and we defined DJC reconstruction to augment UCL reconstruction. To our knowledge, this is the first capsule reconstruction method for thumb MCPJ dorsal capsular tear. Also, the present study had several limitations, including a retrospective nature, a short follow-up period, a small sample size, and no control group.

In conclusion, with encouraging short-term clinical outcomes and a lower complication rate, surgical reconstruction of both UCL and DCL seems to be a safe and effective surgical technique in the management of chronic UCL tears with volar subluxation. This technique should be considered as an alternative treatment method to obtain a stable MCPJ in managing such cases.

## Figures and Tables

**Table 1.** Demographic Characteristics of the Study Participants

Number of patients	7
Gender (female/male)	1/6
Age (year), mean (min–max)	31 (20-39)
Follow-up (month), mean (min-max)	15.5 (12-20)
Right/left wrist	5/2
Dominant/nondominant hand	5/2

**Table 2.** Preoperative and Final Follow-Up Clinical Outcomes of the Patients

Variables	Preoperative	Postoperative	*P*
VAS (mean) (min-max)	5.7 (5-8)	0.57 (0-1)	<.001
Quick-DASH (mean) (min-max)	31.8 (27.3-38.6)	3.2 (0–6.8)	<.001
Glickel grading system (mean) (min-max)	6.1 (5-7)	16.8 (14-19)	
Poor	7	-	
Fair	-	-	
Good	-	3	
Excellent	-	4	
MCPJ ROM (°) (mean) (min-max)			
Flexion deficit	10.7 (0-20)	31.4 (25-35)	.034*
Extension deficit	18.5 (10–25)	0 (0-10)	.022*
Key-pinch strength (%) (mean) (min-max)	33.2 (27-37)	10.2 (6-14)	<.001
Hand grip strength (%) (mean) (min-max)	18.8 (15-23)	6.4 (6-14)	<.001

Quick-DASH, the Quick Disabilities of the Arm, Shoulder, and Hand; VAS, Visual Analog Scale; ROM, range of motion; MCPJ, metacarpophalangeal joint.

*P < .05.
